# Chromium-Cobalt Intoxication with Intense Systemic Complications following Total Hip Revision after Per-Operative Ceramic Fracture

**DOI:** 10.1155/2019/4209796

**Published:** 2019-01-27

**Authors:** Paul Lecoanet, Mathias Blangis, Matthieu Garcia, Yohan Legallois, Thierry Fabre

**Affiliations:** CHU de Bordeaux, Place Amélie Raba-Léon, 33000 Bordeaux, France

## Abstract

**Introduction:**

Heavy metal intoxication after arthroplasty is extremely rare but could be lethal*. Case Report*. We report the case of a 69-year-old woman, who presented intense systemic symptoms of chromium-cobalt intoxication after revision of per-operative fractured ceramic components with metal-on-polyethylene. Systemic toxicity occurred a year after surgery and expressed brutally with mostly central neurological symptoms. Chelation associated with revision surgery allowed rapid regression of all symptoms.

**Conclusion:**

Revision of fractured ceramic, even per-operatively, should not be done with metal-on-polyethylene components, in order to avoid potentially lethal metal intoxication.

## 1. Introduction

Heavy metal intoxication after arthroplasty is extremely rare, but a few cases are reported in literature [1–4]. These cases of cobalt toxicity generally result in replacing fractured ceramic parts with metal-on-metal (MoM) or metal-on-polyethylene (MOP) implants, creating a third-body wear. Friction between residual parts of the ceramic and cobalt-chromium implants creates metal debris, releasing into synovial fluid and the bloodstream. This causes elevated blood cobalt levels and systemic toxicity [5]. Most frequent symptoms of cobalt toxicity are peripheral neuropathy, hearing and visual loss, cognitive decline, and thyroid and cardiac toxicity [6]. Impairment of neurological basal ganglia and caudate nucleus was never described before. This case's interest lies in the severity of symptoms and the fast recovery after appropriate treatment.

## 2. Case Presentation

**Figure 1:**
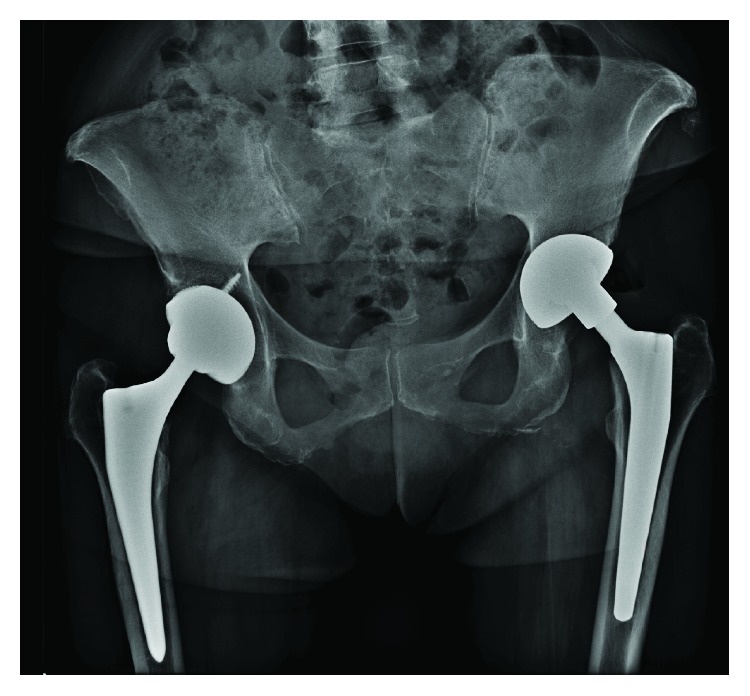
Pelvic A-P view at symptom onset, April 2017. Left periprosthetic collection with hydroaeric levels.

**Figure 2:**
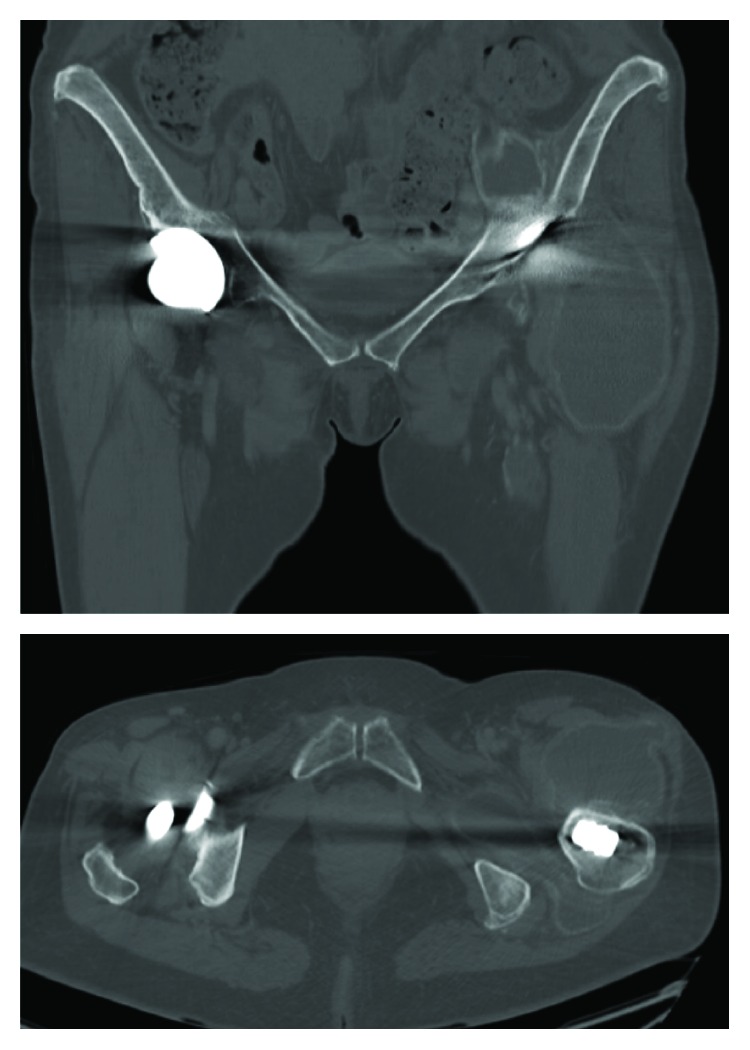
Frontal and axial views of the CT scan showing the important left periprosthetic collection.

**Figure 3:**
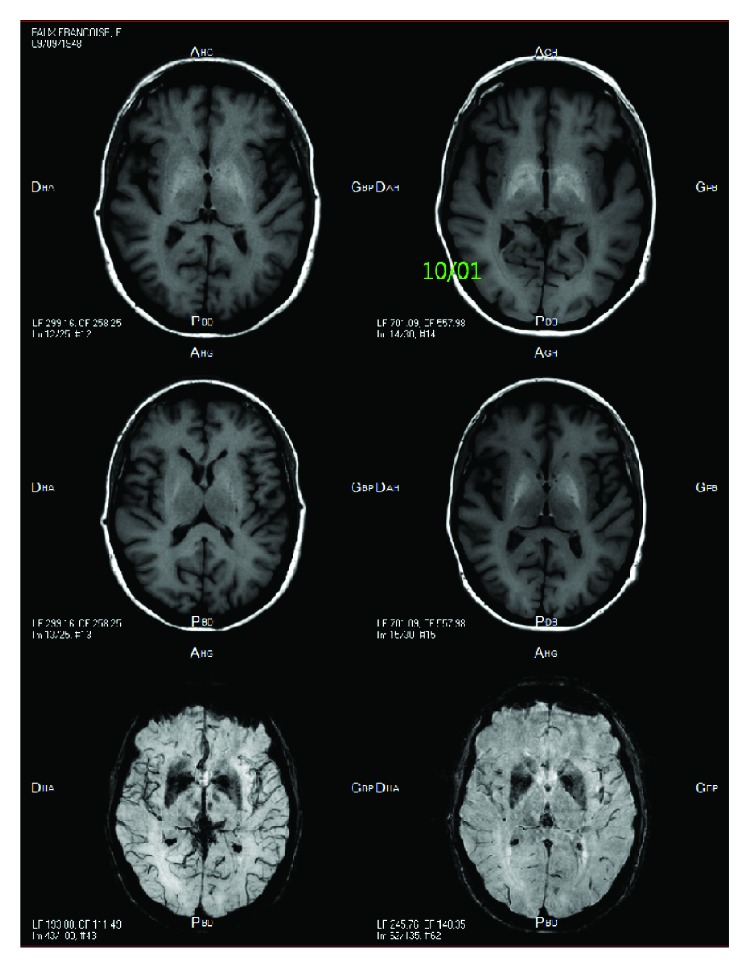
MRI showing the important metal accumulation in the basal ganglia and caudate nucleus with T1 hypersignal and T2 hyposignal.

We report the case of a 69-year-old woman without significant history, who suffered from chromium-cobalt intoxication following total hip arthroplasty (THA). This patient underwent primary arthroplasty to the left hip in 2013 in another orthopedic department, with ceramic bearing. Postoperative course was uncomplicated until 2016 when she experienced three dislocations. Unipolar revision surgery for instability was performed, with per-operative ceramic acetabular liner fracturing in order to remove it. Dual-mobility cup with metal-on-polyethylene components was then implanted. Less than a year after revision surgery, the patient presented to our hospital with fever, asthenia, tachycardia, weight loss, and left groin pain. First clinical and biological evaluation suggested prosthetic joint infection. Plain radiographs and CT scan showed massive collection around the left prosthesis (Figures 1 and 2). A few days later, the patient reported rapid cognitive decline with behavioral disorders, lack of memory, and brutal hearing and visual loss with worsening of asthenia and weight loss (15 kg in 6 weeks due to decreased oral intake, attributed to dysgeusia with metallic taste). Puncture of the periprosthetic collection was performed, revealing metallosis. Neurological exam with MRI showed heavy metal accumulation in the basal ganglia and caudate nucleus (Figure 3). Ophthalmologic and otologic examinations confirmed metal damages to the eyes with bilateral papilledema and to the ears with sensorineural hearing loss limited to 55 dB.

Heavy metal blood levels revealed huge concentrations of cobalt 24808 nmol/L (normal: 0.3-9) (i.e., 1461 *μ*g/L) and chromium 1268 nmol/L (normal: 1-5) (i.e., 65.9 *μ*g/L). Urinary dosage also revealed explosive levels of cobalt 8234 *μ*g/g of creatinine (normal < 2) and chromium 151 μg/L (normal < 1). Regarding these systemic symptoms of metal toxicity, our patient received immediate calcicodisodic EDTA (ethylenediaminetetraacetic acid) and DMSA (dimercaptosuccinic acid) chelation.

**Figure 4:**
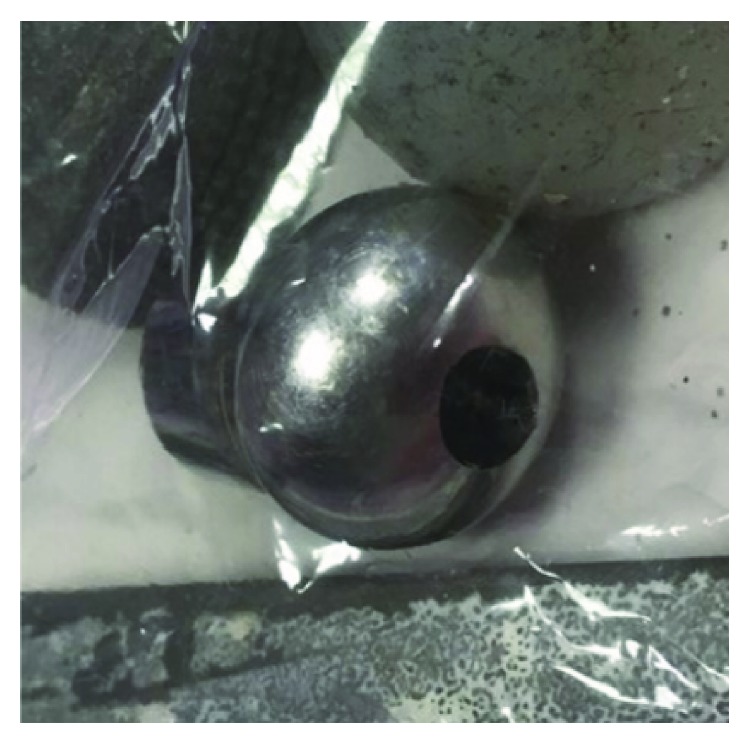
Per-operative aspect of the femoral chromium-cobalt head, flattened with a hole in it. 18 g of metal loss explaining metallosis with systemic repercussions.

One-stage bipolar revision surgery was also performed shortly after, in our institution. Considerable black-liquid collection (around 500 mL, cobalt dosage in liquid > 1 g/L, culture negative for infection) was found around the prosthesis with black staining in periprosthetic tissues and gluteal muscles necrosis. Every component was removed and the cobalt-chromium femoral head was severely worn, with a hole in it (Figure 4). Laboratory exam of the head revealed an 18 g of metal loss, explaining heavy metal intoxication. Ceramic particles around polyethylene and chromium-cobalt head were also found, contributing to metal debris formation. We performed a large and exhaustive synovectomy and debridement of the muscles necrosis and were obliged to implant a dual mobility cup in order to maintain stability of this multioperated hip.

**Figure 5:**
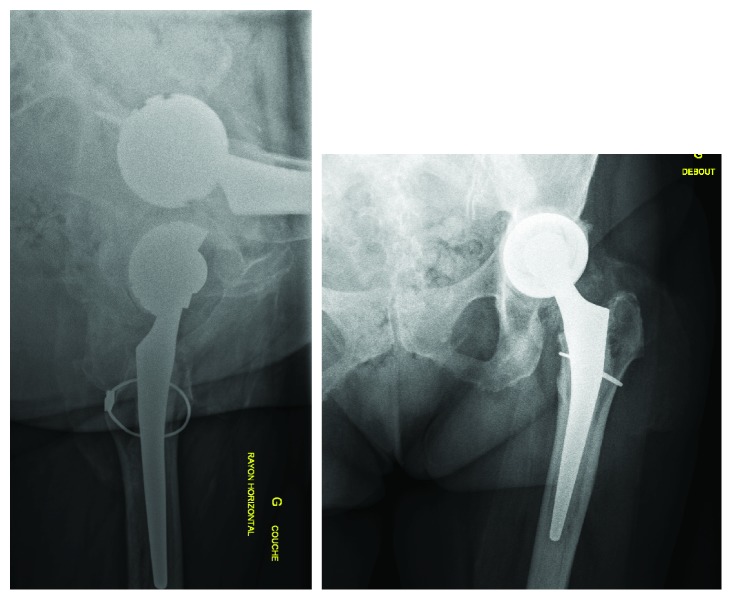
Post revision surgery X-rays at six-month follow-up.

All symptoms along with hip pain rapidly improved after treatment. Cognitive functions were progressively restored. Patient regained audition and vision at functional level. Asthenia, behavioral disorders, and memory loss also disappeared. New MRI examination at 3 months showed reduced T1 hypersignal of the basal ganglia and caudate nucleus. These improvements accompanied a decrease in cobalt and chromium levels. Serum cobalt level went down to 34 and 17 *μ*g/L at, respectively, 1 and 6 months follow-up. Chromium serum levels were at 50 and 40 *μ*g/L at 1 and 6 months. Urinary cobalt level was at 483 *μ*g/g of creatinine and urinary chromium level at 82 *μ*g/L at 6 months. Standard X-rays at the six-month follow-up showed no radiological complications of the left hip revision (Figure 5). Patient could walk without limping or crutches and the Harris Hip Score was 75/100.

## 3. Discussion

In this case of severe metallosis after revision of fractured ceramic components with MOP, neurological repercussions of chromium-cobalt intoxication were put forward, especially, impairment of the neurological basal ganglia and caudate nucleus, as seen on the MRI, that were never described before in this type of case.

Ceramic failure can be usually seen on head component after a traumatic event. In this case, ceramic shattering occurred per-operatively when removing the acetabular liner.

Onset of metallosis systemic symptoms is normally seen within 2 years after revision surgery, and serum levels of cobalt usually ranges from 10 to 2000 times than the normal values [7]. Our patient presented systemic repercussion of metallosis less than a year after revision surgery, with cobalt levels around 3000 times that the normal values. Heavy metal serum levels will be screened on a regular basis, every year for follow-up, as well as clinical and radiological aspects.

Compared to third-body wear from revision of failed ceramic components, toxicity from failed metal-on-metal THAs has been reported to be significantly lower due to the lower levels of cobalt in the blood [8].

Symptomatic cobalt toxicity after revision of failed ceramic component to MOP is well described in literature [7, 9, 10]. After ceramic fracture (although less common since recent advances in manufacturing new generation ceramics [11]), metal-on-polyethylene with synovectomy is often chosen to limit residual ceramic particles and avoid metallosis. Sharma et al. reported a 10-year survivorship in 5 patients if a complete and thorough synovectomy can be performed [12]. However, totally removing all ceramic particles with extensive synovectomy has been proven to be very difficult [13].

## 4. Conclusion

Revision of failed ceramic arthroplasties, even immediate ceramic fracture occurring per-operatively, needs a large synovectomy for removing all ceramic debris and should be done with new ceramic components instead of metal-on-polyethylene, if stability is obtained, in order to avoid metal toxicity, which could be lethal.

## 5. Clinical Message

Cardiomyopathy, visual and hearing loss, or central neurological symptoms appearing after revision of failed ceramic arthroplasty with MOP implants should incite to look for metallosis, even a few years after revision surgery [7].
